# *Mycobacterium microti* Infection in Red Foxes in France

**DOI:** 10.3390/microorganisms9061257

**Published:** 2021-06-09

**Authors:** Lorraine Michelet, Céline Richomme, Edouard Réveillaud, Krystel De Cruz, Jean-Louis Moyen, Maria Laura Boschiroli

**Affiliations:** 1 Paris-Est University, National Reference Laboratory for Tuberculosis, Animal Health Laboratory, Anses, 94700 Maisons-Alfort, France; Lorraine.michelet@anses.fr (L.M.); Krystel.decruz@anses.fr (K.D.C.); 2 Anses, Nancy Laboratory for Rabies and Wildlife, 54220 Malzéville, France; celine.richomme@anses.fr; 3 Regional Directorate for Food, Agriculture and Forest of Nouvelle-Aquitaine, 87000 Limoges, France; edouard.reveillaud@agriculture.gouv.fr; 4 Laboratoire Départemental d’Analyse et de Recherche de la Dordogne, 24660 Coulounieix-Chamiers, France; jl.moyen@dordogne.fr

**Keywords:** red fox, bovine tuberculosis, *Mycobacterium microti*, cross-sectional study

## Abstract

*Mycobacterium microti*, member of the *Mycobacterium tuberculosis*, complex is known to interfere in the screening and diagnosis of bovine tuberculosis. This pathogen is increasingly detected in the frame of surveillance programs for tuberculosis in livestock and wildlife. Recently, red foxes (*Vulpes vulpes*) were found infected by *Mycobacterium bovis* in four French endemic areas. *M. microti* infection was concomitantly found during this investigation. Rates of infection by *M. microti* and *M. bovis* are not different except in one of the four areas (lower prevalence for *M. microti* in Charente). As for *M. bovis* infection, none of the infected foxes presented gross TB-like lesions. Infection of red foxes by *M. microti* seems to occur by ingestion of contaminated food, as mesenteric lymph nodes are mostly infected albeit no fecal excretion could be detected. Red foxes appear to be susceptible to *Mycobacterium microti* infection but seem to play a role of dead-end host for the transmission of this bacillus.

## 1. Introduction

*Mycobacterium microti*, member of the *Mycobacterium tuberculosis* complex (MTBC), was originally described as the agent of tuberculosis (TB) in wild rodents [1]. However, in the last years, an increasing number of cases were described in pets (cat and dog), zoo animals, camelids and, also, in humans [2,3,4,5]. In France, *Mycobacterium microti* interferes with the diagnosis of bovine TB (bTB) and has been identified through the surveillance system in livestock (cattle and goats) [6,7,8] and in wild animals (wild boars and badgers) [2,9]. The presence of this pathogen was also described in wildlife in other European countries [10,11,12,13]. Some authors hypothesize that *M. microti* infection might provide some protection to cattle against *M. bovis* infection (*M. bovis* belonging to the same MTBC), especially in regions where *M. microti* is endemic in badgers and cats [4,14] and could therefore explain the low incidence of bTB in these areas. However, at least at a population level, this protective immunity does not seem to occur in regions where *M. microti* and *M. bovis* infection overlap in some areas in France [8]. Increasing data generated in the recent years—mostly by molecular diagnosis—suggest that the presence of this hard-cultivable pathogen has been underestimated and that the epidemiology of this pathogen, which role as zoonotic agent remains an open question, deserves to be explored.

Bovine TB, mainly due to *M. bovis*, is now considered as a multi-host disease that in Europe affects not only cattle but also wildlife species, such as wild boars (*Sus scrofa*), badgers (*Meles meles*) or wild deer (*Cervus elaphus*, *Capreolus capreolus*). Infection by *M. bovis* has been reported in red foxes (*Vulpes vulpes*) and has been recently identified in four French bTB endemic areas showing prevalence rates similar to those found in badgers in the same areas, raising the question of the role of foxes in the bTB multihost system [15]. During this same study, *M. microti* was also identified in foxes. We present here prevalence values and *M. microti* infection description in this animal species and compare it to that caused by *M. bovis* in these four areas. This is the first report of *M. microti* infection in red foxes.

## 2. Materials and Methods

### 2.1. Ethics Statement

All fox carcasses used in the present study were provided by hunters who held the appropriate permits for hunting foxes under the supervision of local hunting federations, or trappers duly trained and authorized, supervised by pest control officers (historically called wolf-hunter, nowadays being state but volunteer officers in charge of pest control and who supervised trapping) in agreement with national regulations. As the study did not involve invasive procedures on live animals, no ethical approval was necessary.

### 2.2. Study Areas, Fox Sampling and Sample Collection

The study concerns four areas of about 500 km^2^ each in the core of bTB infected areas of Dordogne, Charente and Landes departments, in the South West of France (Nouvelle-Aquitaine region) and of Côte-d’Or department in Eastern France (Bourgogne-Franche-Comté region) [15]. The studied population was the same than in our previous work [15]. Sampling took place from March 2017 to January 2020. In Dordogne, foxes were collected in the whole study area until August 2018 (season #1) and afterwards (season #2) animals from only two bTB hotspots-communes (administrative units) were collected [15]. The expected sample size was determined to enable detection of infection in foxes in each of the four areas, assuming an apparent prevalence of at least 3%, with a 95% confidence interval [15]. Foxes were sent in double packaging to collect points and then to the local laboratories in charge of the necropsies. The following samples were collected at the lab: retropharyngeal (RP) lymph nodes (LN), respiratory LN (composed of tracheobronchial and mediastinal LN), mesenteric LN and any lesion suggestive of tuberculosis.

### 2.3. Detection of M. microti Infection

TB infection was determined by molecular diagnosis performed in (1) a pool of RP LN and respiratory LN, and (2) in the mesenteric LN on animals from the four regions. DNA extraction was performed after mechanical lysis using an LSI MagVetTM Universal Isolation Kit (Life Technologies, Villebon-sur-Yvette, France) with a KingFisherTM Flex automate (Thermo Electron LED S.A.S., Saint-Herblain, France), following the manufacturer’s instructions. For confirming *Mycobacterium tuberculosis* complex (MTBC) the LSI VetMAXTM MTBC Real-Time PCR kit (Life Technologies, Villebon-sur-Yvette, France), which targets IS*6110* and a PCR based on IS*1081* were employed [16]. IS*1561*′ and Rv1510 (RD4) based PCRs were used to differentiate *M. microti* from *M. bovis* infections [16]. Spoligotyping by Luminex, as described by Zhang et al. [17], using TB-SPOL kits purchased from Beamedex^®^ (Beamedex SAS, Orsay, France) on Bio-PLex 200/Luminex 200^®^ was also employed on PCR positive samples for intra-MTBC differentiation and genotyping. The presence or absence of the 43 spacer sequences contained in the DR locus is represented in a binary code of 43 entries. Spoligotypes are named according to an agreed international convention (www.mbovis.org, accessed on 22 October 2020) [18]. Results were interpreted following the manufacturer’s recommendations and by comparison with negative and positive controls.

*M. microti* infection was confirmed when a positive PCR with Rv1510 target and/or a specific *M. microti* spoligotype profile was obtained. With this strategy, and if present, *M. microti* and *M. bovis* coinfection can be identified by IS*1561*′ and Rv1510 concomitant positive PCRs.

Bacterial culture was also performed on the tissues as described previously [15], however they were all negative for *M. microti*.

### 2.4. Data Analysis

For the present study, an infected fox was defined as an animal with an analytical result demonstrating *M. microti* infection by molecular diagnosis. Apparent prevalence in each of the 4 areas and 95% confidence intervals (CI_95%_) were calculated using exact binomial tests. For foxes from Nouvelle-Aquitaine region, the exact Fisher test was used to attest or not of a statistically significant difference of the prevalence in each of the three areas. In each area, *M. microti* prevalence of the present study were compared to *M. bovis* prevalence found in the same samples [15] using the exact Fisher test.

## 3. Results

In Côte-d’Or, the apparent prevalence is 0.7% (CI_95%_: 0.1–3.8%), with one fox found infected by *M. microti* (*n* = 1/146) (Table 1). No infected foxes were identified in Charente (*n* = 0/98), indicating a prevalence lower than 3.7% (upper limit of the 95% confidence interval). In Dordogne (season #1) and Landes, *n* = 12/184 and *n* = 3/140 foxes were found infected, with prevalence values of 6.5% (3.4–11.1%) and 2.1% (0.4–6.11%), respectively. Within Nouvelle-Aquitaine, prevalence in Dordogne is significantly higher than in Charente (*p*-value = 0.0101) and higher than in Landes (with no significant difference, *p*-value = 0.1073).

**Figure 1:**
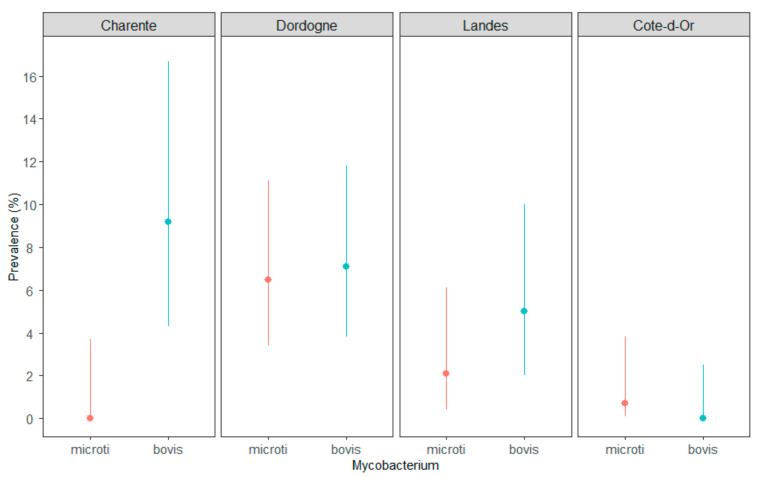
Comparison of *M. microti* (in red) and *M. bovis* (in blue) prevalence in three TB areas of Nouvelle-Aquitaine—Charente, Dordogne (season #1) and Landes—and in the core TB infected area of Côte-d’Or, France. The lines are the CI_95%_ of the prevalence.

The rate of infection by *M. microti* was statistically lower than the rate of *M. bovis* infection in Charente (*p* = 0.0035) but not in Dordogne, Landes or Côte-d’Or (Figure 1).

During season #2 in Dordogne, all *M. microti* infected foxes came from hotspot 1 (Table 2). The rate of infection of foxes infected by *M. microti* was statistically lower than the rate of infected fox by *M. bovis* in hot spot 2 (Fisher exact test, *p*-value < 0.05) whereas no statistically significant difference was observed in hotspot 1.

During season #1, four *M. microti* infected foxes came from hotspot 1 and one from hotspot 2.

**Figure 2:**
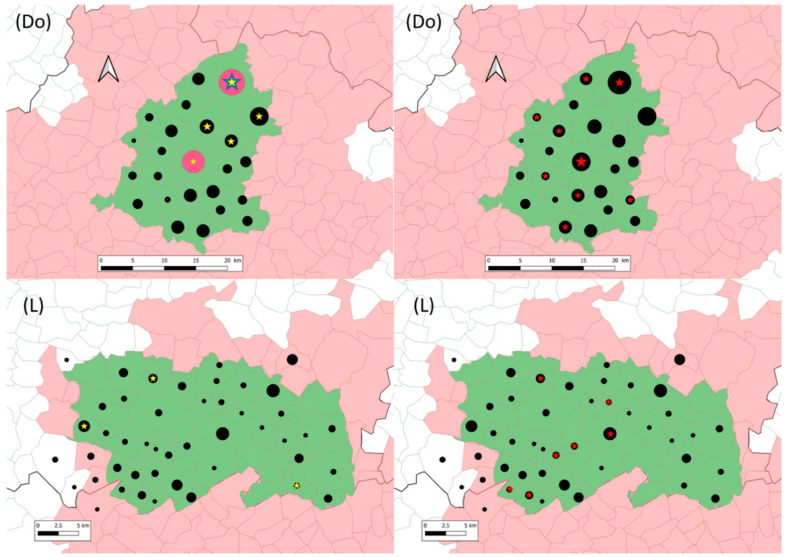
Location of sampled (black dots), *M. microti* infected (yellow (season #1) and blue (season #2) stars) and *M. bovis* infected (red stars) foxes in the Dordogne (Do) and Landes (L) study areas (in green). In Dordogne, red circles represent season #2 sampling in hotspot 1 and 2. The grey lines delimit communes (administrative units). The communes in pink belong to the infected areas where surveillance and management are implemented in cattle and in wildlife species (badgers, wild boars and red deer). Within them, the communes in green are those of included in this study. Black dots and stars are positioned at the center of the communes and their size is proportional to the number of foxes (1 to 27 foxes for dots and 1 to 16 foxes for stars).

In Dordogne still, *M. microti* cases were located in five communes in the northeast of the studied area (Figure 2). Concomitant infection by *M. microti* and by *M. bovis* was observed in two communes. One of these two communes is the bTB hotspot 1, which includes 4 and 12 *M. microti* cases identified in season #1 and season #2, respectively. In Landes, *M. microti* cases were located in three distant communes (Figure 2). In one of them, one *M. bovis* infected fox was also identified.

None of the sampled foxes were identified as co-infected by *M. microti* and *M. bovis*.

In each area, the genotype of *M. microti* that infected foxes was the same as that identified in wildlife species of the same region: SB0118 in Côte-d’Or and Landes, and SB2273 in Dordogne [2].

None of the infected foxes presented gross TB-like lesions. Among the 28 *M. microti* infected foxes during the whole study, 27 (96%) exhibited infection in the mesenteric LN (Table S1), suggesting that the infection was acquired by ingestion of infected food. Fox 180814038269 was positive for the MTBC (IS*6110* and IS*1081* positive PCRs), but intra-MTBC differentiation by PCR (IS*1561*′ and RD4) or spoligotyping was impossible due to a weak DNA concentration. For eight foxes, *M. microti* infection was disclosed in the pool of RP and respiratory LN. Seven of these foxes exhibited detectable *M. microti* infection in the mesenteric LN although for one of them (fox 180814038269) only MTBC was confirmed in this type of LN. A ninth fox (fox 170831039977) was MTBC positive in the pool of RP and respiratory LN and was positive for *M. microti* in the mesenteric LN.

With regards excretion, appropriate samples (swab, urine or kidney and feces) were collected during season #2 in Dordogne for 11 *M. microti* infected foxes. None of these samples was positive for *M. microti* or any other MTBC member.

## 4. Discussion

With the present study, we demonstrated that infection by *M. microti* is prevalent in foxes in areas where bTB is endemic in cattle and where foxes are also found infected by *M. bovis* even at high prevalence [15]. To our knowledge, this is the first work that describes *M. microti* infection in red foxes. However, the absence of previous descriptions does not seem surprising. From the one hand, the recent use of molecular diagnostic tools was able to disclose the wide presence and animal host spectrum of *M. microti*. Indeed, many historical articles describing *M. microti* only used bacteriology, which in the case of this difficult grower has a particularly low sensitivity. On the other hand, and this also stands for *M. bovis*, infection by MTBC does not seem to provoke visible lesions in foxes thus considered negligible actors in bTB epidemiology, and then understudied. The important number of *M. microti* infected foxes found in this study strengthens previous findings showing that *M. microti* is widely spread in the nature [19,20].

Differences in prevalence rates exist between the four studied areas. None or very few *M. microti* cases were identified in Charente, Côte d’Or and Landes, while most of the infected animals were found in Dordogne. For Charente, the absence of cases could be explained by a sampling bias, as the number of sampled animals was lower than in the rest of the studied areas. These results in foxes correlate with those previously found in other wildlife species. In the framework of the TB surveillance program in wildlife in France (Sylvatub) [21], *M. microti* cases were identified in other species in the four areas of this study between 2011 and 2020. In wild boars, one case was found in Charente, another one in Landes and two cases were found in Côte-d’Or. In Dordogne, a larger number of *M. microti* cases were identified: 11 in wild boars and 4 in badgers (Bovine Tuberculosis National Reference Laboratory data).

Foxes have a very large feeding tropism for small rodents, and they have proved to be susceptible to *M. bovis*—thus to MTBC-infection—, therefore, it is not surprising to find them harboring *M. microti* as well. Another means that foxes become infected could be via ingestion of *M. microti*-bearing earthworms (*Lumbricus terrestris*). Indeed, as has been suggested for *M. bovis*, earthworms could act as vectors of mycobacterium present in contaminated environments [22], red foxes being great consumers of these annelids [23,24]—as are badgers or wild boars. These types of environments could exist if they were contaminated by highly infected voles. Besides, ingestion of wild boar *M. microti*-infected carcasses cannot be also excluded as a means of foxes’ infection.

The highest prevalence of *M. microti* infection in foxes was observed in Dordogne. The landscape in the four studied areas is roughly similar, composed of a mixture of pastures and woods. However, we cannot exclude that one reason for explaining the variation in prevalence rates could be the density of rodents, or even earthworms, as mentioned above, which could be associated to local characteristics of the landscape or the period of sampling (sampling occurred in 2017–2018 for Dordogne and in 2018–2019 for the three other areas).

Contrary to what we observed in *M. bovis* infected foxes, excretion does not seem to be occurring in *M. microti* infection, even when mesenteric lymph nodes, as in *M. bovis* infection, are affected. None of the sampled foxes showed the presence of *M. microti* in excreta, while for five *M. bovis* infected foxes—four of which were infected in the mesenteric lymph nodes—this latter bacillus was detected, suggesting active excretion for this pathogen [15]. According to our data, foxes do not seem to play any role in the active transmission of *M. microti*, and would likely be playing that of dead end hosts. However, the number infected foxes found in our study is relatively small and this hypothesis deserves to be confirmed with a larger sample.

Almost all red foxes disclosed *M. microti* infection in mesenteric lymph nodes, suggesting infection by ingestion of contaminated food. European badgers and red foxes can occupy similar ecological niches and can compete over food, including small vertebrates [24]. One of the main preys of red foxes’ diet in Western Europe are field voles (*Microtus agrestis*) [25,26]. Wild mammalian carnivores are consequently likely to have substantial exposure to *M. microti* both from ingestion and their contaminated environment [19]. The differences in prevalence rates by area observed in our study are probably related to the infection and/or presence of the main reservoir, although no data about infection in voles to confirm or refute this hypothesis are available. Indeed, very few data exist on prevalence of this disease in wild rodents. In 2002, 8% of field voles, bank voles (*Clethrionomys glareolus*) and wood mice (*Apodemus sylvaticus*) were found to be infected in England [1]. In another study, prevalence varied between 0% and 50% of infected voles, with an apparent seasonality at the English and Scottish border [27].

Although we found that in some localizations *M. microti* or *M. bovis* presence was exclusive, cohabitation of the two MTBC species could be observed at least at a population level in bTB high burden areas in Dordogne (bTB hotspot 1). Until present, co-infection in a same animal was not demonstrated here or in any other French animal [2,8,9]. For two foxes, 170831039977 and 180814038269, we could not confirm the mycobacterium identity due to a low DNA concentration. However, given that *M. microti* was confirmed in another lymph node of the same animal, it is very probable that the same mycobacterium gave rise to PCR positive results. These observations may be in favor of the hypothesis that *M. microti* infection might provide some protection against *M. bovis* infection at individual level and vice versa.

This study confirms the need of an accurate surveillance of TB using specific molecular tools that overcome the particular difficulty of confirming *M. microti* by bacteriology. Further studies are needed to better characterize the epidemiology of this mycobacteria, to identify the maintenance species and the infection prevalence within their populations as well as climatic, ecological and landscape factors explaining its occurrence and the possible consequences for animal and human public health need to be further explored.

## Figures and Tables

**Table 1:** *M. microti* infection of foxes in three TB areas of Nouvelle-Aquitaine and in the core TB infected area of Côte-d’Or, France.

	Analyzed	Number of Infected Foxes	Prevalence in % (IC_95%_)
Charente	98	0	<3.7 *
Dordogne season #1	184	12	6.5 (3.4; 11.1)
Landes	140	3	2.1 (0.4; 6.11)
Côte-d’Or	146	1	0.7 (0.1; 3.8)

* Upper limit of the 95% confidence interval.

**Table 2:** Results of *M. microti* and *M. bovis* detection in foxes in Dordogne (season #2) and in the two bTB hot spots of this area.

		Number of Infected Foxes (% [CI_95%_])		
	Analyzed	M. microti *	M. bovis **	*p*-Value of the Fisher Exact Test
bTB Hot spot 1	45	12 (26.6 (14.6–41.9))	6 (13.3 (5.0–26.8))	0.3
bTB Hot spot 2	50	0 (0 (0–7.1))	6 (12 (4.5–24.3))	0.0283
Total Dordogne #2	95	12 (6.5)	12 (6.5)	1

* Number of *M. microti* infected foxes in the two hot spots is statistically different (Fisher exact test, *p*-value = 0.0003); ** data from [15].

## Data Availability

The data presented in this study are available in supplementary material Table S1.

## Supplementary Materials

The following are available online at https://www.mdpi.com/article/10.3390/microorganisms9061257/s1, Table S1: Results of *M. microti* detection by molecular diagnosis in tissues and feces, when available, for each of the 28 foxes found infected.
