# Supplementary material for: Mycobacterium microti Infection in Red Foxes in France
Source: Microorganisms. 2021 Jun 9;9(6):1257. doi: 10.3390/microorganisms9061257 (PMC8227042; doi:10.3390/microorganisms9061257)
Supplement: Supplementary file 1 [file microorganisms-09-01257-s001.zip › microorganisms-1232945-supplementary.pdf]

Table S1. Results of *M. microti* detection by molecular diagnosis in tissues, when available, for each of the 28 foxes found infected. LN: lymph nodes; RP: retropharyngeal; resp: respiratory. NR: not reported. MTBC : PCR positive for IS*6110* and IS*1081*, but negative for intra-MTBC PCR (IS*1561*' and RD4)

ID fox	Dept	Season	Date of capture	Sex	Age	LN mesenteric	LN RP + resp	Feces
21 19 REN 1158	Côte-d'Or		29/08/2019	NR	Juvenile	POS	POS	-
170410017992	Dordogne	#1	10/03/2017	NR	NR	POS	NEG	NEG
170523024904	Dordogne	#1	10/05/2017	Female	Adult	POS	POS	-
170606026621	Dordogne	#1	31/05/2017	NR	NR	POS	NEG	NEG
170628030558	Dordogne	#1	23/06/2017	Male	Adult	POS	NEG	NEG
170816037525	Dordogne	#1	04/08/2017	Male	Adult	POS	POS	NEG
170816037531	Dordogne	#1	30/07/2017	Female	Adult	POS	NEG	NEG
170818037900	Dordogne	#1	04/08/2017	Male	Adult	POS	NEG	NEG
170823038432	Dordogne	#1	16/08/2017	Male	Adult	POS	NEG	NEG
170829039569	Dordogne	#1	09/08/2017	Female	Adult	POS	NEG	NEG
170829039575	Dordogne	#1	11/08/2017	Female	Adult	POS	NEG	NEG
170831039977	Dordogne	#1	07/12/2017	Male	Adult	POS	MTBC	NEG
171220055780	Dordogne	#1	26/11/2017	Male	NR	POS	POS	NEG
180524023863	Dordogne	#2	14/05/2018	Female	Adult	POS	NEG	-
180524023865	Dordogne	#2	NR	NR	NR	POS	NEG	-
180814038268	Dordogne	#2	07/08/2018	Male	Adult	POS	NEG	-
180814038269	Dordogne	#2	07/08/2018	Male	Juvenile	MTBC	POS	-
190802033482	Dordogne	#2	05/08/2019	Female	Juvenile	POS	NEG	-
190802033483	Dordogne	#2	05/08/2019	Male	Adult	POS	NEG	-
190805033807	Dordogne	#2	05/08/2019	Female	Adult	POS	NEG	-
190805033803	Dordogne	#2	11/08/2019	Female	Juvenile	POS	NEG	-
190802033486	Dordogne	#2	11/08/2019	Female	Adult	POS	POS	-
190805033804	Dordogne	#2	11/08/2019	Male	Adult	POS	NEG	-
190417017390	Dordogne	#2	22/08/2019	Male	Adult	POS	NEG	-
190823035959	Dordogne	#2	22/08/2019	Male	Adult	POS	NEG	-
0238126	Landes		10/11/2018	Female	Juvenile	POS	POS	-
2589434	Landes		14/11/2018	Male	Adult	POS	POS	-
199041	Landes		27/12/2018	Male	NR[4]	POS	NEG	-
